# Dendrobium Nobile Lindl. Alkaloid Suppresses NLRP3-Mediated Pyroptosis to Alleviate LPS-Induced Neurotoxicity

**DOI:** 10.3389/fphar.2022.846541

**Published:** 2022-05-02

**Authors:** Dai-Di Li, Hong-Xia Fan, Rong Yang, Ying-Ying Li, Feng Zhang, Jing-Shan Shi

**Affiliations:** ^1^ School of Pharmacy, Shanghai University of Traditional Chinese Medicine, Shanghai, China; ^2^ Key Laboratory of Basic Pharmacology of Ministry of Education and Joint International Research Lab of Ethnomedicine of Ministry of Education, Zunyi Medical University, Zunyi, China

**Keywords:** Alzheimer's disease, Dendrobium nobile Lindl. alkaloid, neuroinflammation, NLRP3, pyroptosis, neurotoxicity

## Abstract

Alzheimer’s disease (AD) is a neurodegenerative disorder recognized as a global public health priority. Although available treatments temporarily relieve the symptoms, they could not prevent the progression of cognitive decline. Natural compounds have been rich sources for drug discovery. Dendrobium nobile Lindl. alkaloid (DNLA) is the main active compound in Dendrobium nobile Lindl, a traditional Chinese herbal medicine. Recent studies indicated that DNLA produced neuroprotection. However, the mechanisms underlying DNLA-generated neuroprotection remain unknown. To investigate neuroprotection and the underlying mechanisms of DNLA, mouse hippocampus injection of lipopolysaccharide (LPS)-induced neuronal damage was performed. DNLA protected hippocampus neurons and working memory disorder against LPS-induced neurotoxicity. In addition, DNLA suppressed cell undergoing membrane lysis and cell swelling and inhibited the essential mediator of pyroptosis GSDMD-N expressions. Furthermore, DNLA-mediated neuroprotection was dependent on the inhibition of NLRP3 inflammasome activation, as evidenced by the fact that DNLA reduced pro-inflammatory factor (IL-18 and IL-1β) production and inhibited the expression of related proteins. DNLA-exerted neuroprotection against LPS-induced neuronal damage, and cognitive impairment was not observed in NLRP3 knockout mice. Together, this study suggested that DNLA attenuated NLRP3-mediated pyroptosis to generate neuroprotection against LPS-induced neuronal damage and cognitive impairment.

## Graphical Abstract

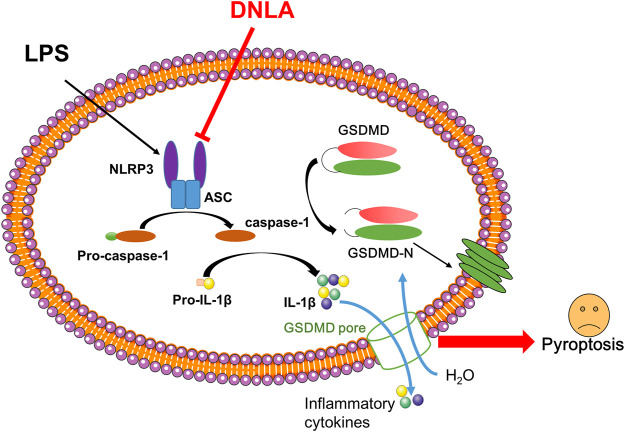

## Introduction

Alzheimer’s disease (AD) is one of the most common neurodegenerative diseases and has affected more than 50 million people worldwide (Lane et al., 2018). The primary neuropathological hallmarks of AD are extracellular neuritic plaques massed by β-amyloid (Aβ) and intra-neuronal neurofibrillary tangles (NFTs) aggregated by hyperphosphorylated tau protein (Kocahan and Dogan, 2017). However, the aforementioned hypothesis should be reconsidered with the failure of numerous clinical trials aiming directly at these hypotheses (Cummings et al., 2019). Recent evidence demonstrates that neuroinflammation plays an important role in the progression of AD (Shi et al., 2020). In the brains of Alzheimer’s patients, aggregation and activation of microglia and astrocytes as well as association with the production of pro-inflammatory factors have been found near misfolded and aggregated proteins. Therefore, inflammation mediated by the immune system might be the key to slowing or preventing the progression of AD (Regen et al., 2017; Dong et al., 2019).

Production of interleukin-1β (IL-1β) is primarily dependent on activation of the inflammasome, a multi-protein complex of cytosolic proteins, such as NOD-like receptor family 3 (NLRP3), apoptosis-associated speck-like protein (ASC), and pro-caspase-1 (Lamkanfi and Dixit, 2014). NLRP3 is the most widely studied inflammasome and mainly expressed in microglia and probably responsible for microglia activation (Eren and ÖZÖRen, 2019). The activation of caspase-1 and subsequent release of IL-18 and IL-1β following the formation of an inflammasome might play a major role in pro-inflammatory responses around microglia (Bauernfeind et al., 2009). Current studies demonstrated that Aβ and NFTs could promote the activation of the NLRP3 inflammasome pathway in microglia, and NLRP3 deficiency attenuated neuroinflammation and Aβ accumulation in the AD mice model (Yin et al., 2017; Ising et al., 2019).

Recent evidence demonstrated that activation of inflammasome pathways triggered pyroptosis and led to the extracellular release of inflammatory cytokines (Liu et al., 2016). Pyroptosis is a kind of inflammatory cell death regulation that relies on the activation of cytosolic inflammasomes, mainly depending on the caspase family (Bergsbaken et al., 2009). Gasdermin D (GSDMD) is recognized as the pyroptotic executive protein, and caspase-1 may mediate the cleavage of downstream GSDMD (Gao et al., 2018). In terms of the pathogenic mechanism of AD, growing investigations have shown that pyroptosis plays an essential role in the occurrence and progression of AD. Moreover, the expression of GSDMD in cerebrospinal fluid of patients was increased, suggesting that it could be employed as a biomarker for AD diagnosis and differentiation from vascular dementia (Shen et al., 2021).

Natural compounds have been rich sources for drug discovery. Dendrobium nobile Lindl. alkaloid (DNLA) is the main active compound in Dendrobium nobile Lindl, a traditional Chinese herbal medicine, which has anti-aging, life-span extension, and immunomodulatory effects (Nie et al., 2020). Substantial studies indicate that DNLA might be beneficial to improve cognitive dysfunctions in AD animal models. The underlying mechanisms were closely associated with reducing the production of extracellular amyloid plaques and regulation of tau protein hyper-phosphorylation (Nie et al., 2018; Liu B. et al., 2020). In addition, DNLA could inhibit lipopolysaccharide (LPS)-induced inflammation and activation of NLRP3 inflammasome in BV2 microglia (Liu H. et al., 2020; Liu J. J. et al., 2020). Whether the neuroprotective effects of DNLA were related to its anti-inflammatory actions was not clear.

In this study, we investigated the effects of DNLA on NLRP3 inflammasome activation and neuron pyroptosis. We further tested the hypothesis that DNLA could alleviate pyroptosis by inhibiting the NLRP3/GSDMD signaling pathway.

## Materials and Methods

### Animals and Treatment

Eight-week-old wild-type C57BL/6 (WT) male mice and male NLRP3 knockout (NLRP3^−/−^) mice on the C57BL/6J genetic background mice were purchased from the Jiangsu ALF Biotechnology Co., Ltd (Nanjing, China). Our laboratory extracted DNLA from Dendrobium nobile stem parts. According to LC–MS/MS, the alkaloids accounted for 79.8%. The chemical structures and the chromatograms of DNLA were shown in our latest articles (Nie et al., 2016). DNLA was dissolved in 1% Tween 80 and was orally administered to each group daily, and the equal volume of vehicle was used as control. WT and NLRP3 KO mice received intragastric administration with DNLA (20 or 40 mg/kg) once daily for 14 days. On the seventh day, the mice were injected with LPS (2 μg in 1 μL normal saline, Sigma-Aldrich, United States) in the hippocampus on both sides of the brain with the following Bregma coordinates: AP 0.5 mm, ML 1.0 mm, and DV −2.0 mm; animals in the control group accepted equal volume of saline. All experiments were in strict conformity with the Chinese Guidelines of Animal Care and Welfare, and this study was approved by the Animal Care and Use Committee of Zunyi Medical University (Zunyi, China).

### Y-Maze Test

Behavioral changes in the spontaneous alternation were assessed via the Y-maze test (Liu B. et al., 2020). The mice were placed in the start area and permitted to freely explore the left or right arm of the maze for 5 min, while the sequence of the arm entrances and total numbers of arm choices were monitored and recorded using a camcorder (TopScan, United States). The total number of arm entries and alternation behavior was counted, and the percentage of spontaneous alternation was calculated by the formula [number of alternation/(number of total arm entries −2)] × 100%.

### Novel Object Recognition Test

Before the novel object recognition test, the mice were acclimated to the open field testing arena for 5 min on day 1. On the second day, the mice were allowed freely to explore two identical objects in the open field for 5 min (training period). On the third day, the mice were allowed to freely explore both objects (one of the objects is novel) for 5 min (test period). The time spent in exploring the objects was measured. The location preference (to assess the impact of position on preference) is described as the recognition index, which is calculated by the time taken to explore novel objects/total time taken to explore both objects *100%.

### Immunofluorescence Staining

All mice were sacrificed after behavior tests. Mice brains were fixed with 4% paraformaldehyde, and brain slices (4 μm thick) were prepared and used for staining. The slices were washed in PBS for 5 min three times and then blotted in 5% goat serum for 1 h at room temperature. The slices were incubated with the primary antibodies overnight at 4°C. The primary antibodies used were: rabbit anti-IBA-1 (1:200, Abcam, United Kingdom), rabbit anti-NLRP3 (1:200, Novus Biologicals, United States), rabbit anti-caspase-1, rabbit anti-GSDMD-N, and rabbit anti-NeuN (1:100, Proteintech, China). After being washed in PBS three times, these slices were incubated with secondary antibodies at 37°C for 1 h, and the secondary antibodies used were Alexa Fluor 488-conjugated goat anti-rabbit IgG (1:400, Abcam, United Kingdom) and Alexa Fluor 594-conjugated goat anti-rabbit IgG (1:400, Abcam, United States). Then, these slices were washed three times again and stained with DAPI (Solarbio, China) for 5 min. The results were imaged using a fluorescence microscope (Nikon, Japan).

### Western Blot Assay

Protein from hippocampus tissue was extracted using lysing buffer according to the protocol from the previous study (Liu B. et al., 2020). The protein was quantified using a BCA protein assay kit (Generay, China) after centrifugation (12,000 rpm, 15 min at 4°C). Protein (30 µg) was loaded onto 10% Tris–glycine polyacrylamide gels and then transferred onto the PVDF membrane (Millipore Trading Co., Ltd.). The membranes were soaked in 5% fat-free milk for 2 h at room temperature and then incubated with primary antibodies overnight at 4°C: anti-rabbit -NLRP3 (1:1000, Novus Biologicals, United States), anti-rabbit ASC (1:1000, Proteintech, China), anti-rabbit-Caspase-1 (1:1000, Proteintech, China), anti-rabbit GSDMD-N (1:1000, HuaAn, China), and anti-rabbit GAPDH (1:5000, Proteintech, China). After washing three times, the membranes were incubated with rabbit HRP-conjugated secondary antibody (1:5000, Proteintech, China) for 1 h at room temperature. The intensity of the blots was detected using a chemiluminescence kit and quantified using Image Lab (Bio-Rad, United States).

### Real-Time PCR Assay

The total RNA of the hippocampus tissues was prepared using RNeasy kit, and the detailed steps of RT-PCR were described previously (Liu J. J. et al., 2020). *NLRP3*, *IL-18*, *IL-1β*, *Caspase-1*, and *GAPDH* genes were tested. Accordingly, the gene expression was normalized with GAPDH. The primers are listed as follows Table1:

**TABLE1:** Primers sequence.

Gene	Sequence (5’ to 3’)
*IL-1β*	GCC CAT CCT CTG TGA CTC AT
	AGG CCA CAG GTA TTT TGT CG
*NLRP3*	ATGCTG CTTCGA CAT CTC CT
	AAC CAA TGCGAG ATC CTG AC
*IL-18*	GCC​ATG​TCA​GAA​GAC​TCT​TGC​GTC
	GTA​CAG​TGA​AGT​CGG​CCA​AAG​TTG​TC
*Caspase-1*	CCCCAGGCAAGCCAAATC
	TTG​AGG​GTC​CCA​GTC​AGT​CC
*GAPDH*	AAC TTT GGC ATT GTG GAA GG
	ACA CAT TGG GGG TAG GAA CA

### Scanning Electron Microscope

The hippocampal tissues were dried in an increasing sequence of ethanol (30–100%), placed on aluminum stabs with their treated surfaces facing up, and sputtered with gold in the JEE-4X equipment (Jeol, Japan). The Hitachi-3000N scanning electron microscope (Hitachi High-Tech, Japan) was used for observation.

### Statistical Analysis

Data were presented as mean ± SEM. The significant statistical difference was analyzed using one-way ANOVA by Dunnett’s *post hoc* t-test and two-way ANOVA with the Bonferroni *post hoc* test for comparison between WT and NLRP3-KO mice groups. The differences were considered significant at *p* < 0.05. Analyses were performed using GraphPad Prism (GraphPad Software, Inc., United States).

## Results

### Dendrobium Nobile Lindl. Alkaloid Ameliorated Lipopolysaccharide-Induced Cognitive Impairment

**FIGURE 1:**
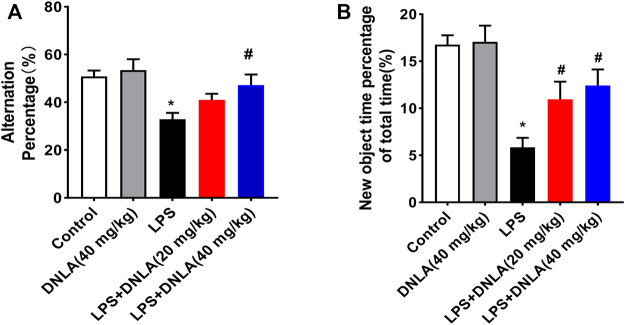
DNLA ameliorated LPS-induced spatial learning and memory function. Wild-type mice were given DNLA (20 or 40 mg/kg) intragastrically for 14 consecutive days. On the seventh day, after DNLA treatment, mice received single intranigral injection of LPS (2 μg) on both sides of the hippocampus. After DNLA for 10 days, the Y-maze test **(A)** and novel object recognition test **(B)** were performed.*p < 0.05 compared with the control group, ^#^ *p* < 0.05 compared with the LPS-treated group. Results were expressed as mean ± SEM, *n* = 10.

Neuroprotective effects of DNLA on the LPS-induced learning and memory function disorder were investigated in WT mice. We analyzed mice learning and memory function changes *via* Y-maze and novel object recognition tests. As shown in the Y-maze result (Figure 1A), LPS reduced the number of alternations compared with the control group (*p* < 0.05), and DNLA improved spatial memory (*p* < 0.05). Similarly, the novel object recognition test indicated that the percentage of time to explore new objects was reduced in the LPS group compared with the control group (*p* < 0.05). However, DNLA treatment increased the percentage of time to explore new objects (*p* < 0.05, Figure 1B). These findings suggested that DNLA improved LPS-induced spatial learning and memory deficits.

### Dendrobium Nobile Lindl. Alkaloid Attenuated Neuronal Damage in Mouse Hippocampus

**FIGURE 2:**
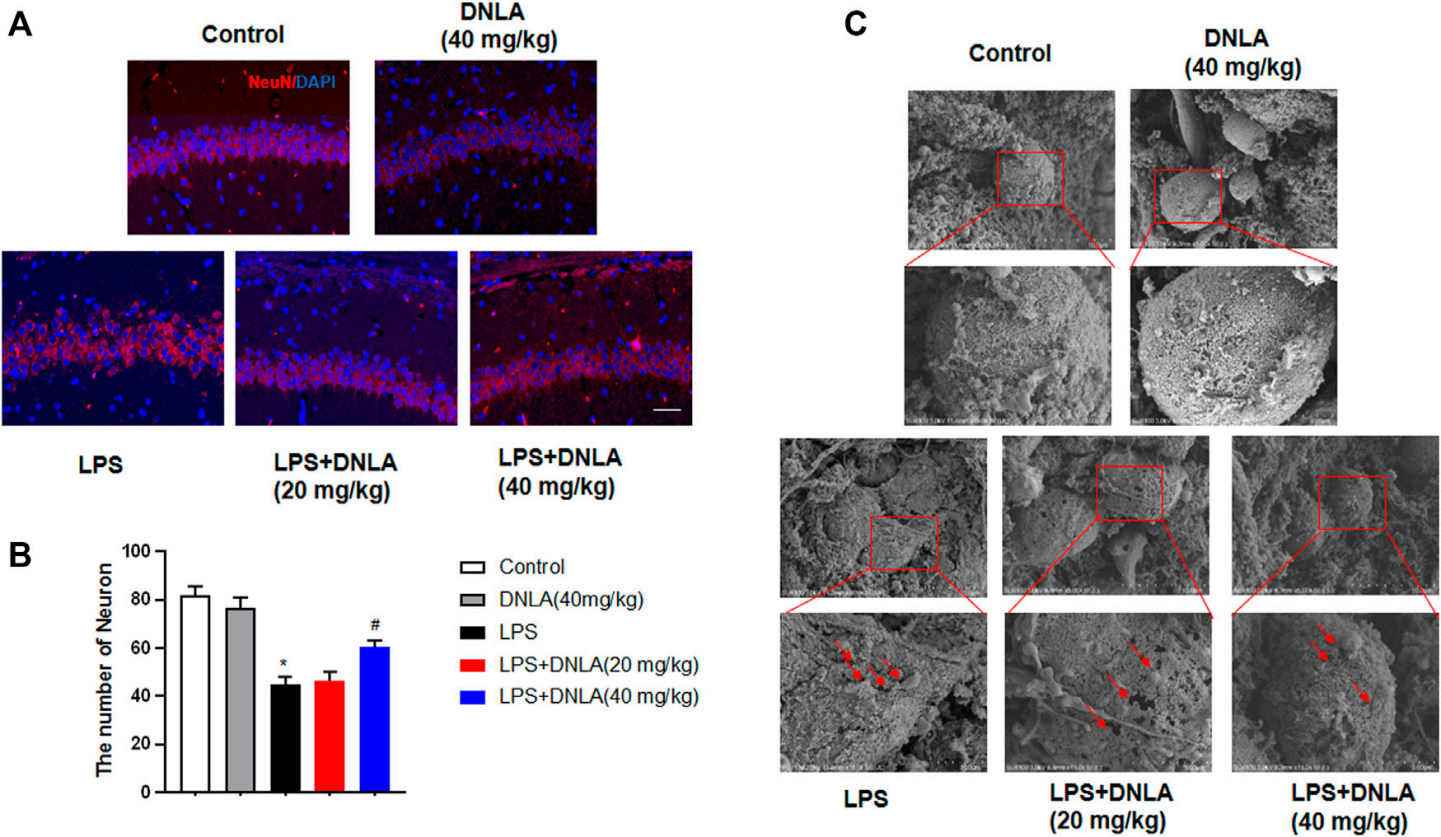
DNLA attenuated LPS-induced hippocampus neuronal damage. After DNLA treatment for 14 days, the neuronal quantification in hippocampus CA1 was measured by immunofluorescence staining. The red color indicated NeuN-positive cells, and the blue color showed a DAPI-stained nucleus. Scale bar = 50 μm **(A)**. Quantitative analysis of the number of NeuN-positive cells **(B)**. Cell membrane structure of hippocampal neurons was observed using the scanning electron microscope **(C)**. *p < 0.05 compared with the control group; ^#^ *p* < 0.05 compared with the LPS-treated group. Data were expressed as mean ± SEM from four mice.

Neuron-specific nuclear protein (NeuN) is a mature neuronal marker, and the number of NeuN-positive cells reflects the growth and development of neurons. As shown in Figures 2A,B, the hippocampus in control and DNLA alone groups was normal with high NeuN-positive immunoreactivity. However, the reduced neuron numbers occurred in the hippocampus CA1 area of the LPS group (*p* < 0.05). DNLA pretreatment protected neurons against LPS-induced neurotoxicity by enhancing the NeuN-positive neuronal number (*p* < 0.05).

Pyroptosis plays an important role in the occurrence of progression of AD. To examine whether pyroptosis affected the neuroprotective effect of DNLA in the WT mice, we assessed the cell membrane structure of hippocampal neurons using the scanning electron microscope. As shown in Figure 2C, the cell membrane of hippocampus neuronal disruption by pores was discerned in the LPS group, and DNLA pretreatment attenuated the LPS-induced neuron membrane damage. These results show that DNLA could generate neuroprotective effect against LPS-induced neurotoxicity.

### Dendrobium Nobile Lindl. Alkaloid Suppressed Microglia and NLRP3 Activation

**FIGURE 3:**
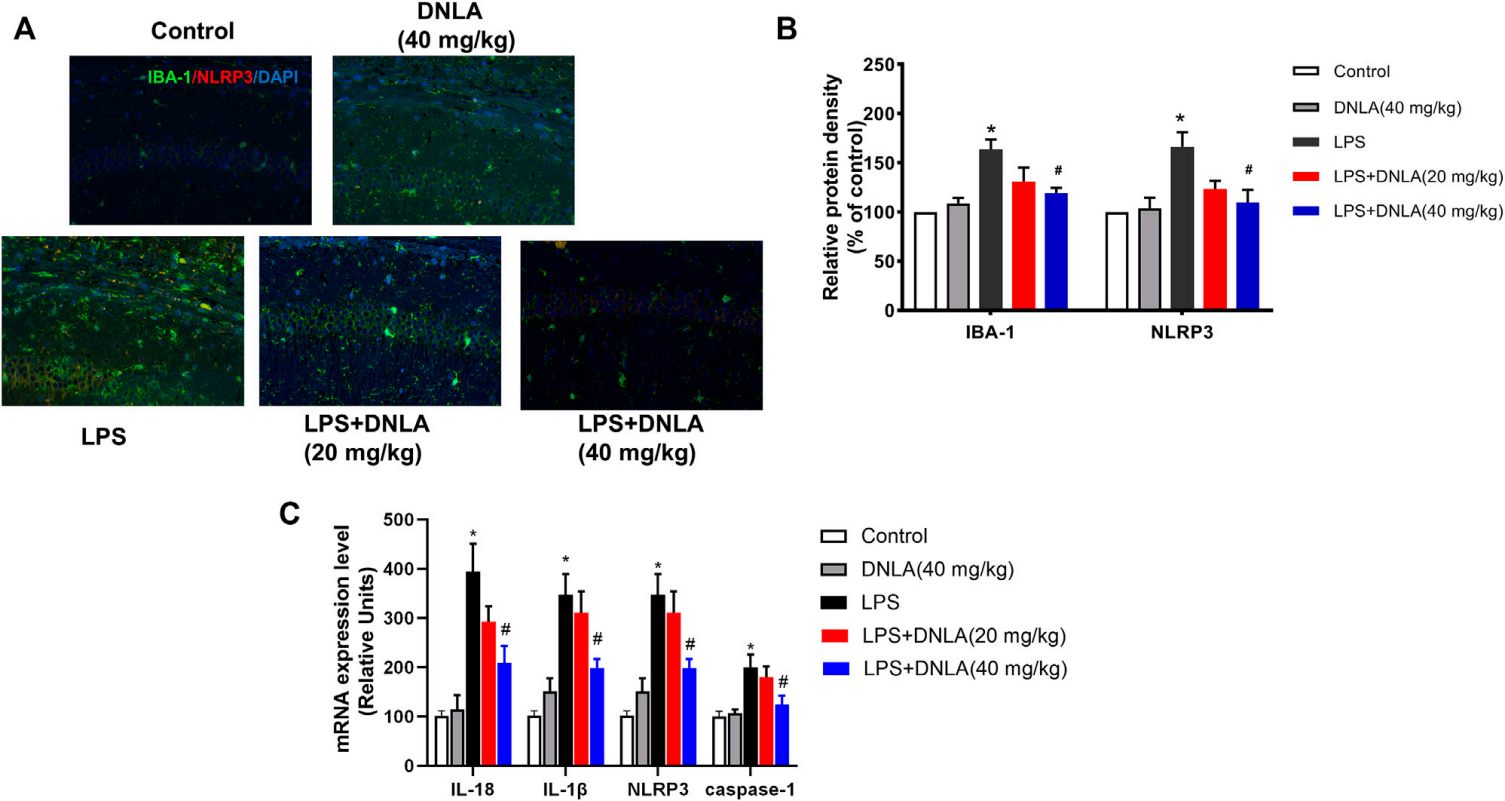
DNLA inhibited microglia and NLRP3 activation. After DNLA treatment for 14 days, the protein expressions of IBA-1 and NLRP3 in the hippocampus CA1 region were measured by immunofluorescence staining. The red color indicated NLRP3, the green color presented IBA-1, and the blue one exhibited a DAPI-stained nucleus. Scale bar = 50 μm **(A)**. Quantitative analysis of protein density of IBA-1 and NLRP3 **(B)**. mRNA levels of NLRP3, IL-18, IL-1β, and caspase-1 in the hippocampus were tested by real-time RT-PCR **(C)**. *p < 0.05 compared with the control group; ^#^ *p* < 0.05 compared with the LPS-treated group. Data were expressed as mean ± SEM, *n* = 4.

**FIGURE 4:**
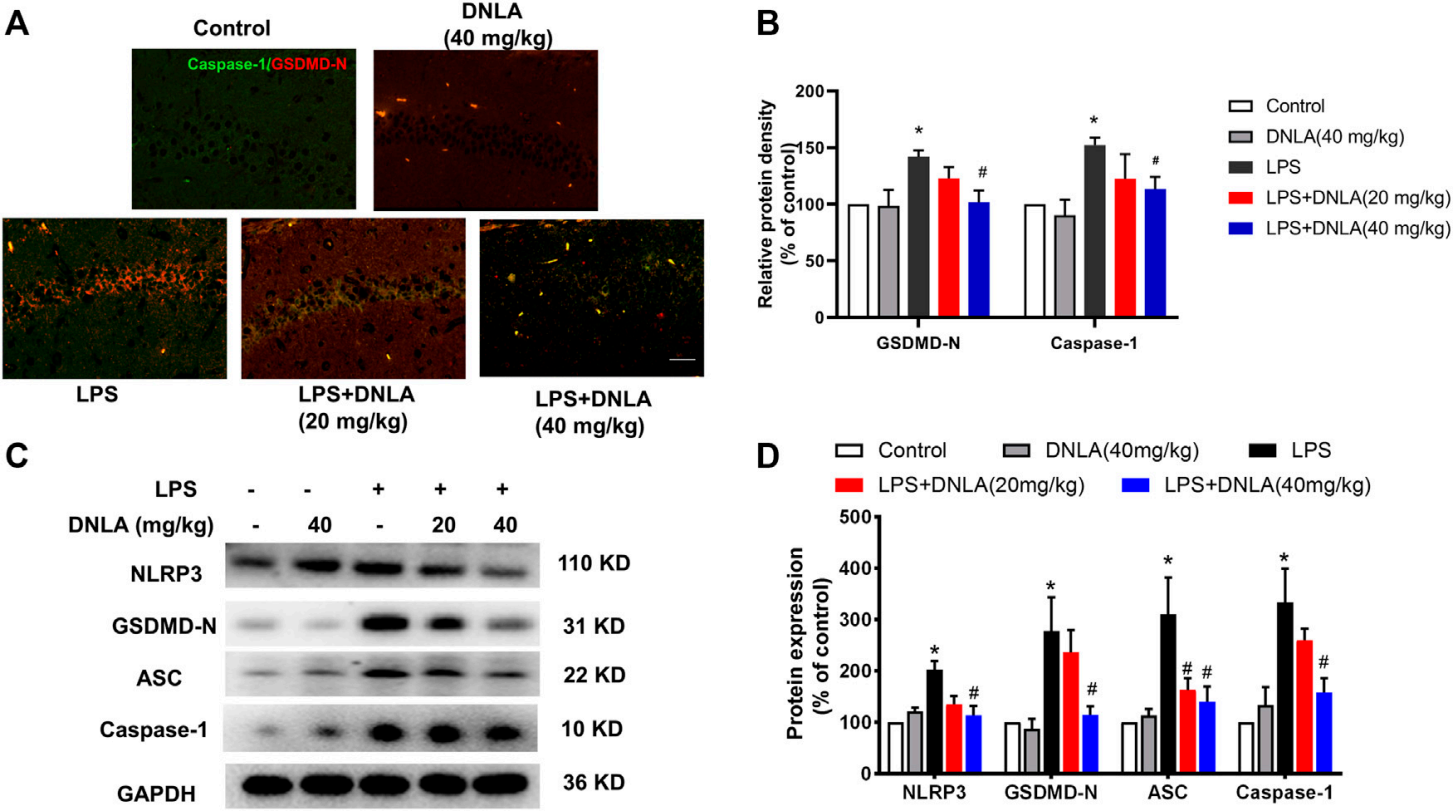
DNLA inhibited NLRP3/GSDMD signaling pathway activation. After DNLA treatment for 14^ ^days, the protein expressions of GSDMD and NLRP3 in hippocampus CA1 were measured by immunofluorescence staining. Scale bar = 50 μm **(A)**. The green color indicated caspase-1-positive cells, and red color exhibited GSDMD-N-positive cells. Quantitative analysis of protein density of GSDMD-N and caspase-1 **(B)**. Protein expressions of GSDMD-N, caspase-1, NLRP3, and ASC in the hippocampus were measured by Western blotting assay **(C)**. Quantitative analysis of the expression of GSDMD-N, caspase-1, NLRP3, and ASC; the reference value of the protein/GAPDH was the ratio of the control group **(D)**. Data were expressed as mean ± SEM, *n* = 3–4. *p < 0.05 compared with the control group; ^#^ *p* < 0.05 compared with the LPS-treated group.

Neuroinflammation is critical in the pathophysiological process of AD. Activation of the NLRP3 inflammasome and microglia plays a key role in the regulation of neuroinflammatory response. To investigate the effects of DNLA on microglia-induced neuroinflammation, the co-localization of microglia and NLRP3 was determined by double immunofluorescence analysis (Figure 3A). In parallel with neuronal damage, LPS induced microglia and NLRP3 activation (*p* < 0.05), and DNLA suppressed these activations (*p* < 0.05, Figure 3B). Furthermore, we revealed that DNLA downregulated the mRNA expressions of NLRP3, caspase-1, IL-1β, and IL-18 in the hippocampus (*p* < 0.05, Figure 3C). Similar results were shown in protein expression of NLRP3, ASC, and caspase-1 in Figure 4A. Together, DNLA treatment attenuated LPS-induced neuroinflammation by inhibiting microglia, NLRP3 inflammasome activation, and release of pro-inflammatory factors.

### Dendrobium Nobile Lindl. Alkaloid Inhibited NLRP3/GSDMD Signaling Pathway Activation

To confirm whether DNLA inhibited the NLRP3/GSDMD signaling pathway in mouse hippocampus, immunofluorescence analysis was performed to determine the expression of caspase-1 and GSDMD-N. As shown in Figures 4A,B, the fluorescence intensity of caspase-1 and GSDMD-N was more prominent after LPS treatment (*p* < 0.05). Compared with the LPS group, DNLA reduced the protein expressions of caspase-1 and GSDMD-N (*p* < 0.05). Moreover, the protein expression of the NLRP3/GSDMD-N signaling pathway was measured by Western blotting; the higher level of GSDMD-N, caspase-1, ASC, and NLRP3 protein expressions was observed in the LPS group (*p* < 0.05), which were inhibited by DNLA treatment (*p* < 0.05, Figures 4C,D).

**FIGURE 5:**
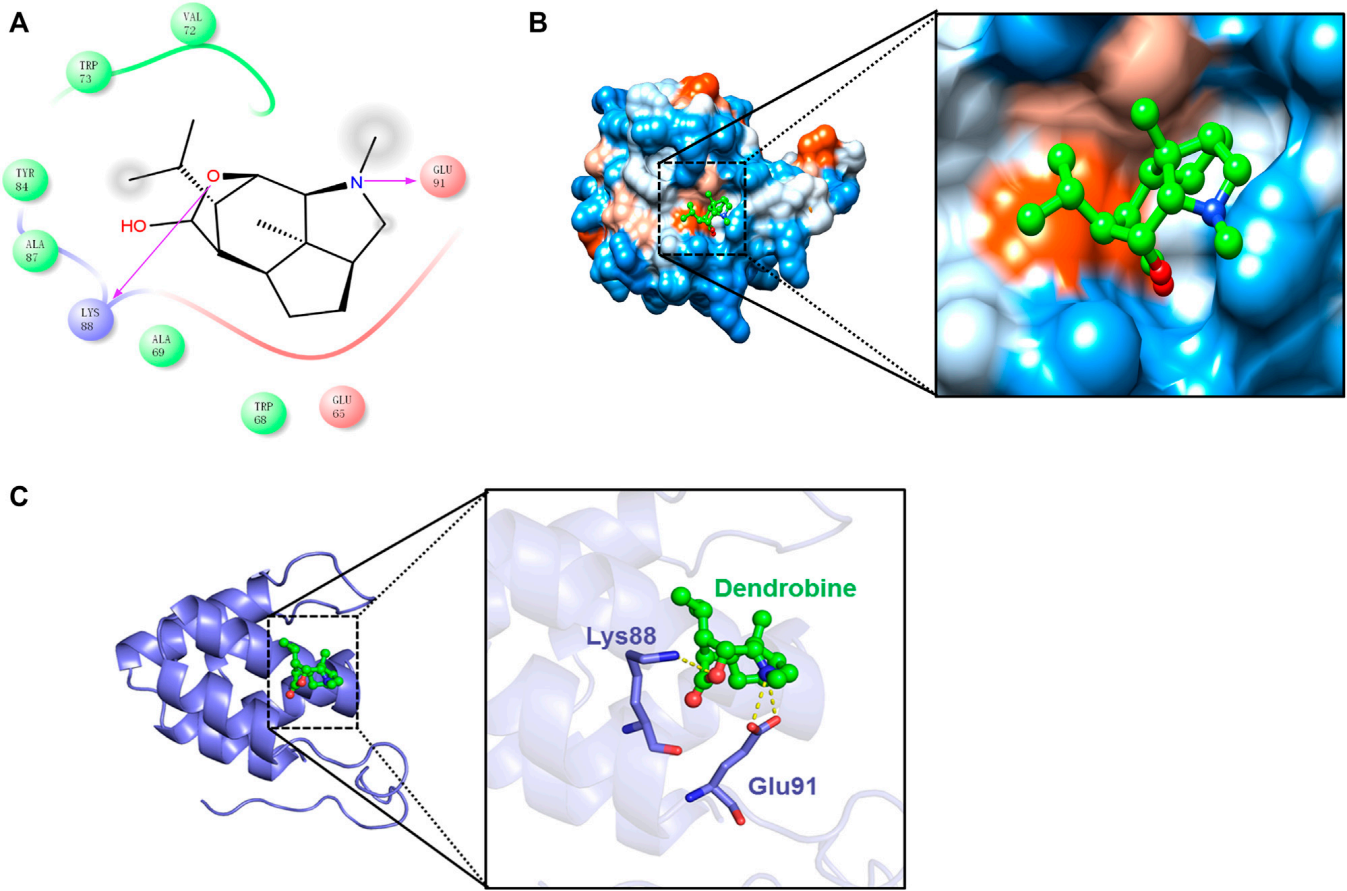
Molecular docking of the DNLA and NLRP3 complex. Residues of amino acids between DNLA and NLRP3 complex **(A)**. Binding of DNLA to the hydrophobic surface of the NLRP3 protein; hydrophilic (blue) and hydrophobic parts (orange) of the protein surface **(B)**. Overview of the predicted binding mode between GSDMD-N and NLRP3 **(C)**.

Additionally, the results further exhibited the strong binding affinity between DNLA and NLRP3 with a binding energy of −6.15 kcal/mol. Thereafter, the presumptive binding modes and the pocket of the amino acid were tested by molecular docking, including Val72, Trp73, Tyr84, Ala87, Glu91, and Lys88, which further confirmed that DNLA bound to the hydrophobic pocket of NLRP3, which is consistent with the results *in vivo* (Figure 5). These findings indicated that DNLA might directly bind to NLRP3 to exert its pharmacological activities.

### NLRP3 Signaling Participated in Dendrobium Nobile Lindl. Alkaloid-Mediated Neuroprotection

**FIGURE 6:**
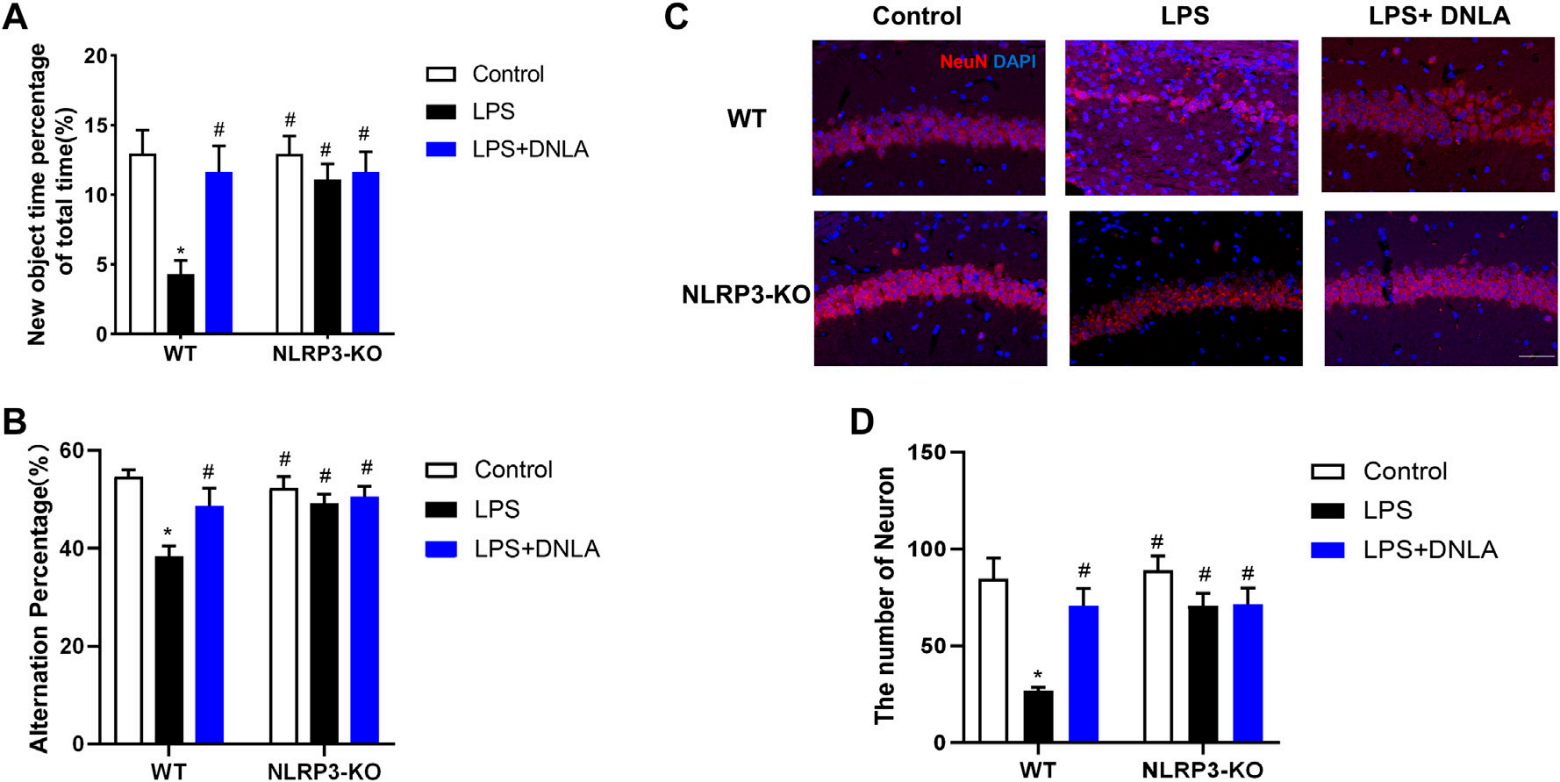
NLRP3 signaling participated in DNLA-mediated neuroprotection. WT mice and NLRP3-KO mice were given DNLA (40 mg/kg) intragastrically for 14 consecutive days. On the seventh day after DNLA treatment, mice received a single intranigral injection of LPS (2 μg) on both sides of the hippocampus. After DNLA treatment for 10 days, the novel object recognition test **(A)** and Y-maze test **(B)** were determined. The neuronal number in hippocampus CA1 was measured by immunofluorescence staining; the red color indicated NeuN-positive cells, and the blue represented the DAPI-stained nucleus. Scale bar = 50 μm **(C)**. Quantitative analysis of the number of NeuN-positive cells **(D)**.*p < 0.05 compared with the WT control group; ^#^ *p* < 0.05 compared with the WT LPS-treated group.

**FIGURE 7:**
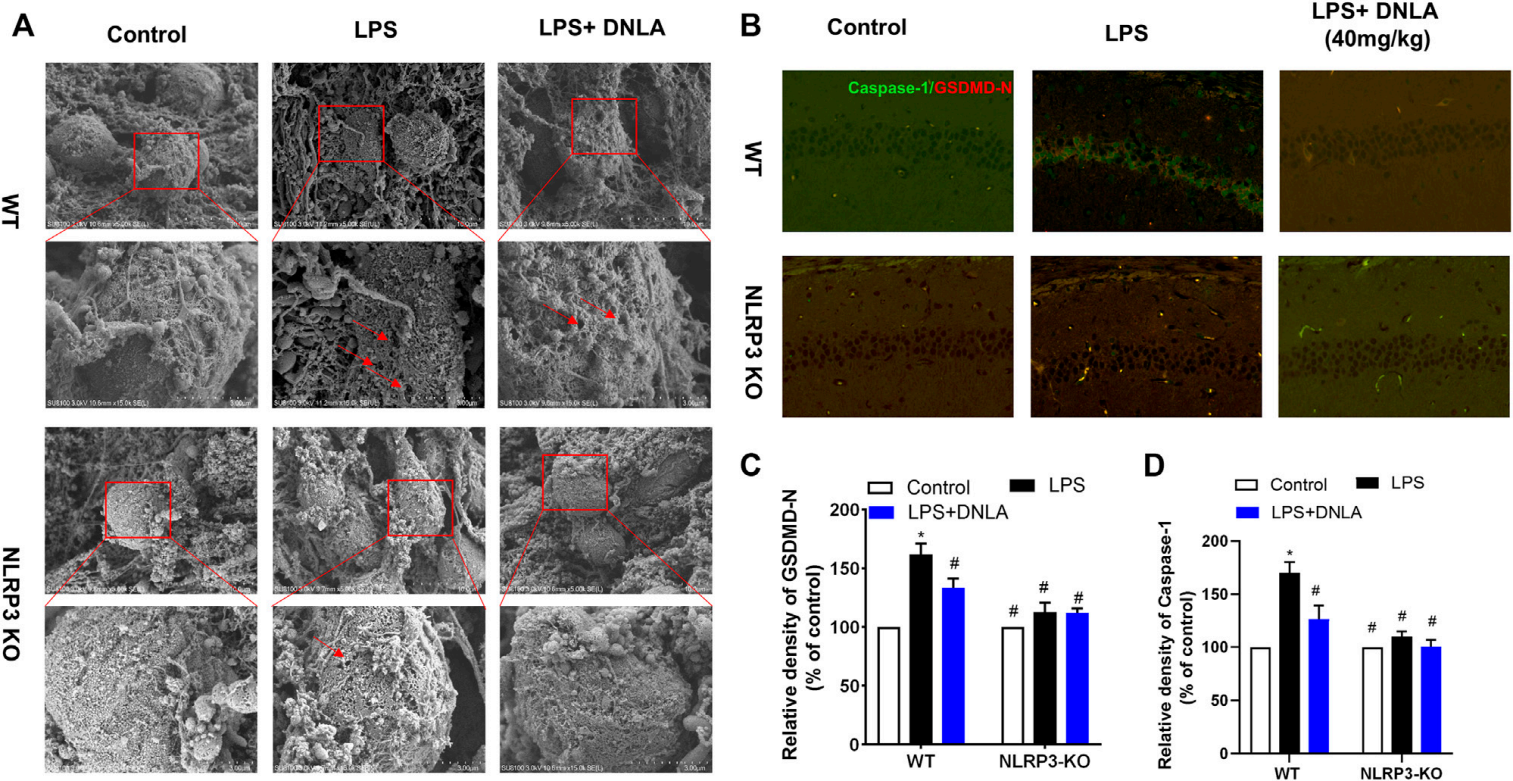
DNLA suppressed pyroptosis through inhibition of NLRP3 signaling. After DNLA treatment for 14 days, the cell membrane structure of the hippocampal neurons was discerned using the scanning electron microscope **(A)**. Protein expressions of caspase-1 and NLRP3 in hippocampus CA1 were measured by immunofluorescence staining. The green color indicates caspase-1-positive cells, and red color indicated GSDMD-N-positive cells. Scale bar = 50 μm **(B)**. Quantitative analysis of protein density of GSDMD-N **(C)** and caspase-1**(D)**. *p < 0.05 compared with the WT control group; ^#^ *p* < 0.05 compared with the WT LPS-treated group. Data were expressed as mean ± SEM (*n* = 3).

Since DNLA ameliorated NLRP3 inflammasome activation, the neuroprotection effect of DNLA was further evaluated by using NLRP3 KO mice. First, the DNLA-ameliorated LPS-induced learning and memory function disorder was not discerned in NLRP3 KO mice (Figures 6A,B). In addition, DNLA-mediated neuroprotection was absent in NLRP3 KO mice via NeuN immunostaining in the hippocampus. In detail, as shown in Figure 6C, LPS injection caused neuronal death in WT mice, while DNLA-attenuated neuronal loss in NLRP3 KO mice appeared to be less evident than that in WT mice. Similar results were shown in the cell membrane structure of hippocampal neurons using the scanning electron microscope (Figure 7A). Furthermore, DNLA reduced LPS-induced protein expressions of caspase-1 and GSDMD-N in WT mice, whereas DNLA-mediated reduction expressions of caspase-1 and GSDMD-N were not discerned in NLRP3 KO mice (Figures 7B–D). Collectively, these results suggested that inhibition of NLRP3 expression participated in DNLA-mediated neuroprotection in mice hippocampus.

**FIGURE 8:**
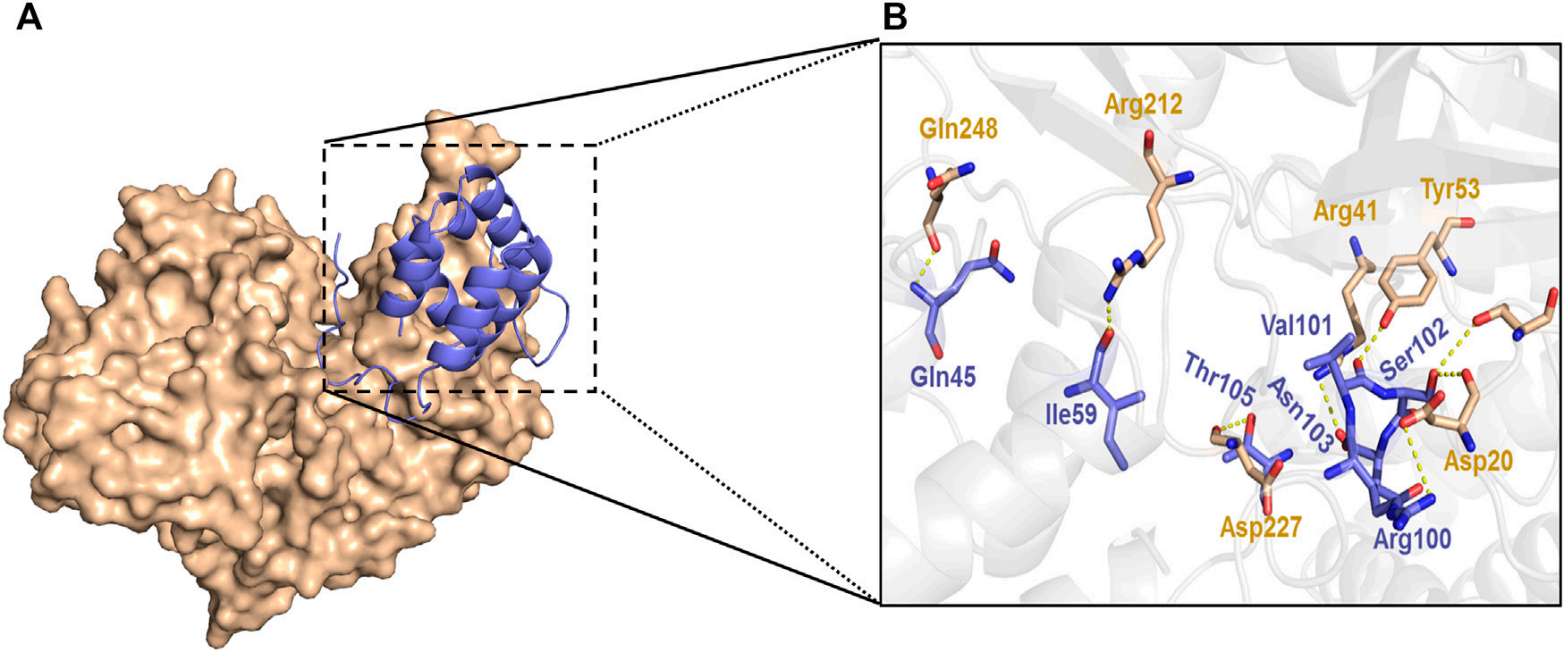
Interaction between NLRP3 and GSDMD-N. Interaction between NLRP3 and GSDMD-N was displayed using ZDOCK. Substrate-binding surface **(A)**. Interactions of NLRP3 with active amino acid sites and GSDMD-N **(B)**. The NLRP3 protein is shown in purple color solid ribbon, while GSDMD-N protein is in yellow.

The results further demonstrated that knockdown of NLRP3 attenuates GSDMD-N expression. Furthermore, the docking of NLRP3 and GSDMD-N was carried out by the ZDOCK method same as the previous study. The results showed that there were strong interactions between NLRP3 and GSDMD-N as evidenced by the output value score of 1330.175 (Figure 8). Of note, the results further demonstrated that hydrogen bond and charge interactions were generated through the amino acid residues including Gln45, Ile59, Arg100, Val101, Ser102, Asn103 and Thr105 in NLRP3 and Asp20, Thr22, Arg41, Tyr53, Arg212, Asp227, and Gln248 in GSDMD-N. These findings suggested that there existed a potent affinity between NLRP3 and GSDMD-N.

## Discussion

We previously demonstrated that DNLA improved cognitive deficits in several AD animal models (Yang et al., 2014; Nie et al., 2018; Liu B. et al., 2020). Here, the current investigation found that LPS-induced NLRP3 inflammasome activation caused neuronal injury in a pyroptotic-dependent way. DNLA inhibited the activation of the NLRP3 inflammasome and the subsequent release of pro-inflammatory cytokines such as IL-1β and IL-18 in the hippocampus. Furthermore, DNLA protected LPS-induced neuronal injury in hippocampus and working memory impairment. Collectively, these findings revealed that NLRP3-mediated pyroptosis played a detrimental role in the etiology of LPS-induced memory impairment, and DNLA acted as a promising therapeutic agent for the prevention and treatment of AD.

It is well-known that the hippocampus is responsible for learning and spatial memory, while acute inflammation, oxygen deficit, or other cause damage to the hippocampus (Wu et al., 2015). Those acute damages are selectively sensitive in the hippocampal CA1 region neurons and cause cell death, whereas the cortical, dentate gyrus (DG), and CA3 regions appear to be more resistant (Bartsch et al., 2015; Bartsch and Wulff, 2015). Thus, we investigated neuronal injury in the hippocampal CA1 region and discovered that mice exposed to LPS had significantly damaged neurons in this region. Moreover, mice exposed to LPS performed worse in the Y-maze test and novel object recognition test than those in the control group, indicating that spatial learning and memory dependent on the hippocampus were impaired.

In addition, neuroinflammation is a pathological feature of AD (Webers et al., 2020; Leng and Edison, 2021). Several studies revealed that inflammatory processes might promote neuronal loss and cognitive decline (Calsolaro and Edison, 2016; Bradburn et al., 2019). LPS, an inflammation inducer, has been confirmed to influence Aβ deposition, and LPS injection to the mouse brain ventricle caused memory deficiency and Aβ accumulation (Park et al., 2020). Another evidence clearly demonstrated that DNLA treatment protected rat brains from LPS-induced neuroinflammation via inhibiting microglia and NLRP3 activation.

Studies revealed that activation of the NLRP3 inflammasome in the hippocampus might cause neuronal damage, cognitive dysfunction, and even neuronal death (Hanslik and Ulland, 2020). NLRP3 inflammasome is a protein complex and consists of NLRP3, ASC, and pro-caspase-1, leading to the cleavage of caspase-1 and the maturation and secretion of IL-1β and IL-18 (Chen et al., 2019; Stancu et al., 2019). To estimate the effects of DNLA against LPS induced-NLRP3 inflammasome pathway activation of hippocampus, we analyzed gene expression of the principle members of the NLRP3 inflammasome pathway in the hippocampus and found that DNLA downregulated the mRNA expressions of NLRP3, caspase-1, IL-1β, and IL-18 in mouse hippocampus.

Several preliminary studies proved that the NLRP3 inflammasome accelerated activation of caspase-1. Caspase-1 could cleave GSDMD into the GSDMD-N domain, which further forms pores on lipid membranes and induces cell swelling (Fan et al., 2018; Li et al., 2020). Pyroptosis, a caspase-1-mediated form of cell death, is characterized by an early breakdown of the integrity of plasma membranes that leads to an extracellular spill of intracellular pro-inflammatory cytokines (Sun et al., 2019). Like apoptotic cell death, pyroptosis is another mode of programmed cell death associated with the cellular inflammatory response. Pyroptosis plays a crucial part in immune defense against body damage, such as stroke and infection (Liu H. et al., 2020). When cells are damaged, damage-associated molecular patterns (DAMPs) initiate the inflammatory response by binding to DAMP receptors, such as TLRs. For infection, the microbial molecules with DAMPs initiate the response by binding to the corresponding PRR (Humphries et al. ., 2020). Following this, the cell undergoes membrane lysis and cell swelling, and eventually the membrane ruptures leading to the release of the pro-inflammatory contents. In this study, we found that DNLA inhibited IL-1β release and pyroptosis of hippocampal neurons.

In addition, a large number of studies confirmed that GSDMD is a candidate for pyroptotic pore formation and the key contributor to pyroptosis (Humphries et al., 2020). As a result, we hypothesized that GSDMD was essential for the effect of DNLA on hippocampal pyroptosis. This investigation discovered that DNLA reduced LPS-induced GSDMD enhancement as well as caspase-1 expression. Furthermore, we demonstrated that several pores were formed on neuronal membranes, and GSDMD-N was upregulated in the hippocampus exposed to LPS, which was reversed by DNLA.

At present, nutraceuticals have become new promising compounds for preventive and beneficial for AD. DNLA is the main active compound in Dendrobium nobile Lindl. As reported, substantial preclinical studies indicate that DNLA is a promising molecule to counteract various pathophysiological processes of AD, by improving cognitive functions and inhibiting neurodegeneration. The underlying mechanisms might be related to inhibition of the production of Aβ plaques and Tau protein hyper-phosphorylation as well as by suppressing neuroinflammation and apoptosis and activating autophagy (Li et al., 2011; Li et al., 2017). However, the molecular mechanism of DNLA inhibiting neuroinflammation complex formation remains elusive. This study demonstrated that DNLA produced neuroprotection through the amelioration of NLRP3-mediated pyroptosis (Graphical Abstract). Nevertheless, we used lateral ventricle injection with LPS to induce neuroinflammation, study the mechanism of neuroinflammation in hippocampal neuron damage, and learn about memory defect, but this is far from the clinical progression of AD patients. Therefore, DNLA-mediated neuroprotection still warrants the exploration in other animal models, such as 3XTg or 5XTg AD mice, and also further comprehensive clinical investigations are needed to elucidate the multiple practical and theoretical issues of DNLA-generated neuroprotection in AD.

## Conclusion

This study demonstrated that DNLA suppressed neuronal pyroptosis induced by LPS, and these beneficial effects were closely associated with the inhibition of NLRP3/GSDMD pathway activation.

## Data Availability

The original contributions presented in the study are included in the article/Supplementary Material, further inquiries can be directed to the corresponding authors.
